# An Anti-Inflammatory Azaphenothiazine Inhibits Interferon β Expression and CXCL10 Production in KERTr Cells

**DOI:** 10.3390/molecules23102443

**Published:** 2018-09-24

**Authors:** Leon Strzadala, Anna Fiedorowicz, Edyta Wysokinska, Ewa Ziolo, Małgorzata Grudzień, Malgorzata Jelen, Krystian Pluta, Beata Morak-Mlodawska, Michal Zimecki, Wojciech Kalas

**Affiliations:** 1 Institute of Immunology and Experimental Therapy, Weigla 12, 53-114 Wroclaw, Poland; strzadal@iitd.pan.wroc.pl (L.S.); anna.fiedorowicz@iitd.pan.wroc.pl (A.F.); edyta.wysokinska@iitd.pan.wroc.pl (E.W.); ziolo@iitd.pan.wroc.pl (E.Z.); malgorzata.grudzien@iitd.pan.wroc.pl (M.G.); zimecki@iitd.pan.wroc.pl (M.Z.); 2 Department of Organic Chemistry, School of Pharmacy with the Division of Laboratory Medicine, The Medical University of Silesia, Jagiellońska 4, 41-200 Sosnowiec, Poland; manowak@sum.edu.pl (M.J.); pluta@sum.edu.pl (K.P.); bmlodawska@sum.edu.pl (B.M.-M.)

**Keywords:** azaphenothiazine, anti-inflammatory activity, CXCL10, KERTr cells, keratinocytes, IFNβ, TLR3

## Abstract

An azaphenothiazine derivative, 6-chloroethylureidoethyldiquino[3,2-b;2′,3′-e][1,4]thiazine (DQT), has recently been shown to exhibit immunosuppressive activities in mouse models. It also inhibited the expression of CXCL10 at the protein level, at non-toxic concentrations, in the culture of KERTr cells treated with double-stranded RNA, poly(I:C). In this report, we demonstrated that DQT inhibits the transcription of the CXCL10 gene. Although CXCL10 is an IFNγ-inducible protein, we found that the CXCL10 protein was induced without the detectable release of IFNγ or IκB degradation. Hence, we concluded that IFNγ or NFκB was not involved in the regulation of the CXCL10 gene in KERTr cells transfected with poly(I:C), nor in the inhibitory activity of DQT. On the other hand, we found that IFNβ was induced under the same conditions and that its expression was inhibited by DQT. Kinetic analysis showed that an increase in IFNβ concentrations occurred 4–8 h after poly(I:C) treatment, while the concentration of CXCL10 was undetectable at that time and started to increase later, when IFNβ reached high levels. Therefore, DQT may be regarded as a new promising inhibitor of IFNβ expression and IFNβ-dependent downstream genes and proteins, e.g., CXCL10 chemokine, which is implicated in the pathogenesis of autoimmune diseases.

## 1. Introduction

Phenothiazines are compounds exhibiting various biological activities [1]. Classical phenothiazines with aminoalkyl substituents serve as a source of valuable drugs [2]. Modification of phenothiazines with azine rings results in the formation of azaphenothiazines [3]. Recently, we have synthesized a series of azaphenothiazines by replacing benzene rings with pyridine and quinoline rings [4,5,6,7,8]. Some of these compounds significantly inhibited mitogen-induced proliferation of human peripheral blood mononuclear cells, tumor necrosis factor alpha (TNFα) production in human whole blood cultures and the growth of tumor cell lines. Recent studies have also shown that newly synthesized azaphenothiazine derivatives exerted suppressive activity in in vivo models of: delayed-type hypersensitivity to ovalbumin and cutaneous carrageenan reaction [9], contact sensitivity to oxazolone [10] and experimental psoriasis in mice [11].

We found in previous studies that low concentrations of 6-chloroethylureidoethyldiquino[3,2-b;2′,3′-e][1,4]thiazine (DQT) inhibited CXCL10 protein synthesis in KERTr cells treated with synthetic double-stranded RNA (mimicry of RNA virus infection) [11]. CXCL10 chemokine is an IFNγ-inducible 10-kDa protein [12] implicated in the pathology of many autoimmune diseases through the recruitment and activation of T cells, neutrophils and monocytes. CXCL10 expression is increased in such autoimmune disorders as psoriasis, diabetes, rheumatoid arthritis, systemic sclerosis, systemic lupus erythematosus, Sjögren’s syndrome and idiopathic inflammatory myopathy (for a review, see [13]). Therefore, we studied the anti-inflammatory activity of DQT in normal human keratinocytes to find the mechanism of CXCL10 expression inhibition by DQT. This may be important because this recently synthesized azaphenothiazine derivative may serve as a novel inhibitor of CXCL10 chemokine secretion with potential efficacy in therapy of autoimmunological diseases [4,9,10,11].

## 2. Results

### 2.1. DQT Compound Inhibits CXCL10 Release upon Induction with Poly(I:C)

**Figure 1:**
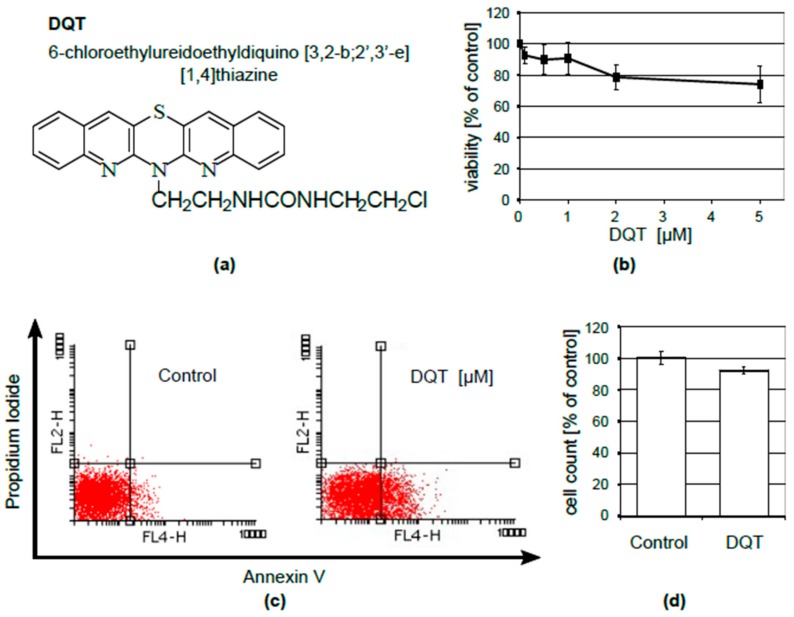
6-Chloroethylureidoethyldiquino(3,2-b;2′,3′-e)(1,4)thiazine (DQT) compound has negligible toxicity to KERTr cells. (**a**) Chemical structure of the azaphenothiazine derivative, DQT. (**b**) Viability of KERTr cells treated with DQT for 24 h, tested by the MTS assay. The results are presented as a percentage of the untreated control. Mean values and standard deviations of five independent experiments are shown. (**c**) Representative dot plots of Annexin V and propidium iodide staining of KERTr cells treated with 5 μM DQT for 24 h; the percentage of Annexin V-positive cells (early apoptosis) is shown. (**d**) Cell counts after 24-h treatment of KERTr cells with 5 µM of DQT; the results are presented as a percentage of the untreated control; mean values of three independent counts are shown.

Recently, DQT (Figure 1a) has been presented as a potential therapeutic in psoriasis [11]. CXCL10 has been identified as one of its functional targets in a normal human keratinocyte cell line, KERTr.

**Figure 2:**
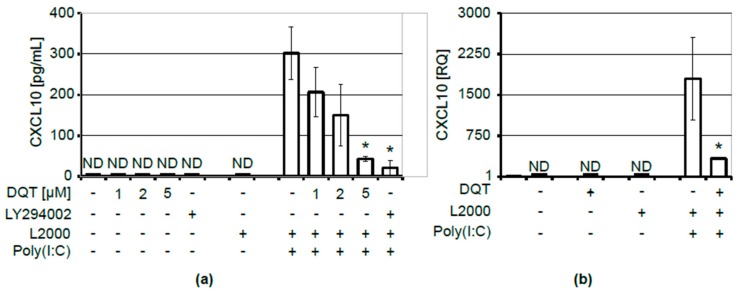
DQT compound inhibits the poly(I:C)-induced production of CXCL10 at the transcriptional level; (**a**) CXCL10 protein levels in cell culture media after 24 h of transfection with 0.2 µg of poly(I:C) using Lipofectamine 2000 (L2000) and subsequent DQT or 25 µM LY294002 treatment; mean values and standard deviation of three independent experiments are shown; the results below showing the sensitivity range of the ELISA assay are marked as ND (not detected); statistical significance is marked with asterisks; (**b**) relative quantification of CXCL10 mRNA expression assayed by real-time RT-PCR in conditions described in (**a**); for inhibition of CXCL10 mRNA expression, cells were treated with 5 µM DQT; mean values and standard deviations of two individual experiments are shown.

Although CXCL10 produced by untreated human keratinocytes was undetectable in culture medium, its release was induced by lipid-facilitated transfection of KERTr cells with poly(I:C) synthetic RNA that targets intracellular TLR3 receptors. We found that the poly(I:C)-induced production of CXCL10 by KERTr cells was significantly reduced by DQT treatment (Figure 2a). DQT decreased CXCL10 levels in a concentration-dependent manner, wherein 1 μM of DQT was sufficient to reduce CXCL10 release (Figure 2a).

DQT might exert its effect on CXCL10 concentrations in the culture medium either by influencing CXCL10 protein expression/secretion processes or by impeding its transcription. We analyzed changes in CXCL10 mRNA levels to find which of these mechanisms was involved in the DQT-induced decrease in CXCL10 secretion. We found that the induction of CXCL10 secretion by poly(I:C) required the activation of CXCL10 gene expression, which was drastically reduced by DQT treatment (Figure 2b). Thus, DQT regulated CXCL10 expression at the transcriptional level in KERTr cells. Changes in the studied chemokine expressions could not be attributed to DQT toxicity, because 5 µM DQT had a very limited influence on KERTr cell viability, as determined by the MTS assay (Figure 1b). At the same time, we observed a similar increase in phosphatidyl serine externalization (from 7% in control to 26% after 5 µM DQT treatment), with no accompanying impact on cell membrane integrity, as measured by Annexin/PI staining (Figure 1c). Last but not least, no significant effect of DQT on cell number (Figure 1d) was observed.

### 2.2. Inhibition of CXCL10 Release by DQT Is Associated with Its Effect on IFNβ, But Not IFNγ Expression or NFĸB Activation

**Figure 3:**
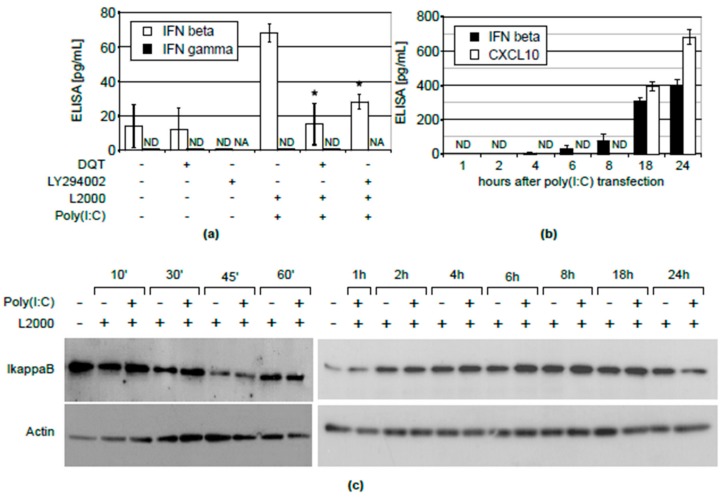
DQT inhibits poly(I:C)-induced CXCL10 synthesis in KERTr cells by inhibiting IFNβ, but not IFNγ production; (**a**) levels of IFNβ and IFNγ in cell culture media of KERTr cells transfected with 0.2 µg of poly(I:C) using Lipofectamine2000 (L2000) and/or treated with 5 µM DQT or 25 µM of LY29002 for 24 h; mean values and standard deviations are shown; IFNγ was undetectable (ND); non-assessed probes are marked as NA; (**b**) kinetics of IFNβ and CXCL10 induction upon transfection with poly(I:C); mean values and standard deviations of two independent experiments are shown; (**c**) Western blot of IκB from KERTr cells transfected with 0.2 µg of poly(I:C) using Lipofectamine2000 (L2000) alone or treated with 5 µM DQT for the indicated time; representative blots are shown.

Data on the regulation of CXCL10 in normal human keratinocytes are scarce. In general, three main pathways involving the IFNγ, INF β and NFκB systems are implicated in the regulation of CXCL10 expression. In the first instance, we found that the stimulation of KERTr cells with poly(I:C) did not lead either to the induction of IFNγ release to cell culture media (Figure 3a) or to activation of IFNγ gene transcription (data not shown). On the other hand, we observed a considerable induction of IFNβ expression upon transfection with poly(I:C) (Figure 3a). The kinetic experiment (Figure 3b) showed that the induction of CXCL10 (at 18 and 24 h) was preceded by IFNβ release, which was detectable as early as 4 h after transfection with poly(I:C). This result suggests that the induction of CXCL10 expression, by activating the TLR3 receptor with poly(I:C), occurs in an IFNβ-dependent manner. Thus, we asked whether the induction of IFN-β by poly(I:C) would be affected by DQT. We found that the treatment with this azaphenothiazine could substantially reduce the induction of IFNβ expression by poly(I:C) (Figure 3a). The same effect was observed when a known inhibitor of the TLR3/4-mediated induction of IFNβ, LY294002 [14], was used. Moreover, similarly, the LY29002 treatment abrogated CXCL10 expression (Figure 2a). These results indicate that the IFNβ pathway is a potential target for DQT’s suppressive action. The NFκB transcription factor can be also implicated in the regulation of CXCL10 expression. The activation of NFκB is reflected in the level of the IκB inhibitory protein. Therefore, we determined changes in the expression of IκB protein upon transfection with poly(I:C) in a wide time range using Western blotting. As in the case of IFNγ, we did not observe any significant changes in IκB levels after poly(I:C) treatment, indicating that NFκB is not involved in the regulation of TLR3-mediated CXCL10 expression in KERTr cells (Figure 3C).

## 3. Discussion

Hitherto, our studies have shown that the azaphenothiazine derivative, DQT, exerts an immunosuppressive effect in in vivo models of delayed-type hypersensitivity to ovalbumin, carrageenan-induced footpad inflammation [9] and contact sensitivity to oxazolone [10]. More recently, we have shown that DQT can act by inhibiting the production of TNF-α, IL-8 and CXCL10 by describing its in vivo anti-psoriatic activity and potential feasibility in therapy [11]. The latter chemokine has been implicated in several autoimmune disorders, as CXCL10 expression is increased in patients with psoriasis [15], rheumatoid arthritis [16], systemic sclerosis [17], systemic lupus erythematosus [18], Sjögren’s syndrome [19] and idiopathic inflammatory myopathy [20]. We decided to study the mechanism underlying CXCL10 induction by poly(I:C) and its inhibition by DQT, because CXCL10 expression is induced in human keratinocytes in active psoriatic plaques [21] and may be suppressed by anti-psoriatic drugs [22]; and because DQT has a strong impact on CXCL10 expression. For this purpose, we used normal human keratinocytes (KERTr cells) activated with synthetic double-stranded RNA, engaging internal TLR3 receptors (mimicry of RNA virus infection). We found that DQT, at low non-toxic concentrations (Figure 1), inhibited CXCL10 expression in a dose-dependent manner, not only at the protein level (Figure 2a), but also at the level of transcription (Figure 2b). CXCL10 gene transcription is regulated by signaling pathways elicited by interferons β and γ, as well as by the NFκB transcription factor, whose activation is controlled by the IκB inhibitory protein [13,23,24]. Therefore, we wanted to determine which of these mechanisms is involved in the inhibition of CXCL10 gene expression by the DQT compound. It appeared that in KERTr cells, only IFNβ was induced by poly(I:C) transfection, but not IFNγ (Figure 3a). Importantly, the induction of IFNβ was inhibited by DQT (Figure 3a). In contrast, the expression of IκB, an inhibitor of the NFκB pathway, was not markedly changed after poly(I:C) treatment (Figure 3c). As the NFκB is the leading transcription factor regulating immune response, a lack of IκB degradation underlines the uniqueness of the keratinocyte cell line model. Hence, we concluded that DQT acts as an inhibitor of IFNβ expression induced by double-stranded RNA in KERTr cells, thereby suppressing subsequent CXCL10 expression. This conclusion is further supported by the results of kinetic analysis, showing that in double-stranded RNA stimulation of normal human KERTr cells, the induction of CXCL10 (at 18 and 24 h) was preceded by IFNβ release, which was detectable as early as 4 h after transfection (Figure 3b). Moreover, the known inhibitor of the TLR3/4-mediated induction of IFNβ, LY29002, down-regulated poly(I:C)-induced expression of both IFNβ and CXCL10. Overall, this suggests that the regulation of CXCL10 by activation of the TLR3 receptor with poly(I:C) occurs in an IFNβ-dependent manner. IFNβ plays diverse and critical roles in human autoimmune disorders. Increased signaling of IFNβ plays a pathogenic role in the aforementioned autoimmune diseases [25,26,27,28]. On the other hand, IFNβ is used as a therapeutic in multiple sclerosis [29], an autoimmune disease of the central nervous system. Such apparently conflicting roles of this cytokine could be a result of tissue- and time-specific IFNβ signaling in epithelial, lymphoid and nervous tissues. Additionally, IFNβ treatment was associated with the occurrence of psoriasis-like skin lesions [30,31,32], showing potential benefits of IFNβ inhibition in maintaining skin homeostasis. However, it should be underlined that DQT inhibits both IFNβ and CXCL10, which play critical pathogenic roles in many autoimmune diseases. We anticipate that it would be interesting to study DQT’s suppressive activity in the models of the above-mentioned autoimmune disorders or, for example, in experimental diabetes in NOD mice.

## 4. Materials and Methods

### 4.1. DQT

6-Chloroethylureidoethyldiquino[3,2-b;2′;3′-e][1,4]thiazine was synthesized as previously described [5,6].

### 4.2. Cell Culture and Treatment

All experiments were performed using KERTr, a human skin keratinocyte cell line (full name: CCD 1106 KERTr) obtained from ATCC. KERTr cells were cultured in keratinocyte-SFM serum-free medium (Gibco, Thermo Fisher Scientific, Waltham, MA USA) with the addition of bovine pituitary extract (Gibco) (0.05 mg/mL), epithelial growth factor (Sigma, Merck KGaA, Darmstadt, Germany ) (35 ng/mL) and antibiotic/antifungal mixture (Sigma). For transfection with poly(I:C) (polyinosinic:polycytidylic acid) or DQT administration, the cells were plated 1 day before treatments, at a density of 105 cells/0.5 mL of medium. Poly(I:C), a synthetic analogue of viral double-stranded RNA, was applied to immunostimulate KERTr, whereas the DQT compound was tested for its anti-inflammatory potential. Before use, DQT was dissolved in DMSO for 1 h at 37 °C. The solution was kept in an ultrasonic bath for 30 min to complete the dissolving process. The cells were transfected in fresh medium without antibiotics. The transfection was performed with 0.2 μg poly(I:C) using the Lipofectamine 2000 Reagent (Invitrogen, Thermo Fisher Scientific), according to the manufacturer’s instructions (2 µL/60 µL). DQT was used at concentrations ranging from 0.2 μM–5 μM, depending on the experiment and LY29002 (Sigma) in a concentration of 25 μM. Cell cultures treated with Lipofectamine 2000 or DMSO were considered as controls for poly(I:C) transfection or DQT applications, respectively. The treatment included: transfection with poly(I:C) and 4 h of incubation, DQT administration to fresh medium, a subsequent 4 h of incubation and medium replacement and final incubation for another 18 h (24 h from transfection time). An alternative treatment involved 4 h of pre-incubation with DQT, transfection with poly(I:C) and a second incubation with DQT. All steps were performed in fresh medium. The kinetics of IFN-β and CXCL10 release was established for: 1-, 2-, 4-, 6-, 8-, 18- and 24-h time points after poly(I:C) transfection. Similarly, the cellular extracts for Western blotting analysis were collected after 10, 30, 45 min, 1, 2, 4, 6, 8, 18 and 24 h after transfection with poly(I:C). Collected media and cellular extracts (for Western blotting or real-time PCR analysis) were frozen at −30 °C until use.

### 4.3. Cell Viability Assay

The MTS colorimetric assay was used to establish DQT cytotoxicity. KERTr cells were plated on a 96-well plate at a density of 5 × 10^3^ cells/100 μL medium and were treated with the DQT compound at a concentration range of 0.2–5 μM. After 24 h, the cells were incubated with MTS containing PMS (CellTiter 96 AQueans Non-Radioactive Cell Proliferation Assay; Promega, Madison, WI, USA) for 2 h at 37 °C. The absorption of the color product was determined spectrophotometrically at 490 nm.

### 4.4. Flow Cytometry

Externalization of phosphatidylserine was detected using the Annexin V-APC apoptotic detection kit (BD Bioscience, BD Pharmingen™) according to the manufacturer’s recommendations. Apoptosis was quantified as the percentage of Annexin V+/PI− and Annexin V+/PI+ cells. Cell suspensions were analyzed on FACSCalibur (Becton-Dickinson, Franklin Lakes, NJ, USA).

### 4.5. Cell Counting

The number of cells was determined using a MOXI Z cell counter (ORFLO, Ketchum, ID, USA).

### 4.6. Western Blotting

Cell extracts were prepared in RIPA buffer, containing 150 mM NaCl, 50 mM Tris-HCl pH 8.0, 1% SDS, 1% NP-40, 0.5% sodium deoxycholate and SigmaFAST protease inhibitor cocktail (Sigma-Aldrich, St. Louis, MO, USA). Protein concentrations were measured using the Pierce BCA Protein Assay Kit (Thermo Fisher Scientific). Ten micrograms of proteins were loaded onto the gel in Laemmli buffer and separated by SDS-PAGE. The separated proteins were transferred to a PVDF membrane (Merck Millipore, Burlington, MA, USA). Non-specific binding sites on the membrane were blocked with 1% casein in TBST (0.1% Tween) for 1 h at room temperature (RT). Then, the membrane was incubated overnight at 4 °C with anti-IκB (1:1000) (Santa Cruz Biotechnology, Dallas, Texas, USA) antibody and/or 1 h at RT with anti-actin-HRP (1:2000) (Santa Cruz Biotechnology) or anti-rabbit-HRP (1:3000) (Dako, Agilent Technologies, Santa Clara, CA, USA) antibodies. HRP-catalyzed reactions were developed using the SuperSignal West Dura Extended Duration Substrate (Thermo Fisher Scientific).

### 4.7. Quantitative Polymerase Chain Reaction (Real-Time RT-PCR)

Total RNA was extracted from the cells using a TRI reagent solution (Invitrogen, Carlsbad, CA, USA). DNase I (Thermo Scientific) was used to remove potential genomic DNA contamination. Total RNA (6 μg) was digested in the presence of RNAse inhibitor (RiboLock RNase Inhibitor, Thermo Scientific) according to the manufacturer’s protocols. An aliquot of 1.5 μg of total RNA was used to make the first strand complementary cDNA (in 20 µL) using the Maxima First Strand cDNA Synthesis Kit (Thermo Scientific), according to the manufacturer’s protocol. Each PCR reaction (20 μL, three replicates in a 96-well plate) contained 10 μL DyNamo Flash Probe qPCR Kit (Thermo Scientific), 1.3 μL of cDNA, 1 μL of each primer (10 μM) and 6.7 μL of H_2_O. GAPDH-VIC (Hs02786624_g1), CXCL10-FAM (Hs00171042_m1), IFNγ-FAM (Hs00989291_m1) and IFNβ-FAM (Hs0027188_s1) were used for qPCR analysis (Thermo Fisher Scientific). qPCR was conducted using the Applied Biosystems ViiA 7 Real-Time PCR System (Thermo Fisher Scientific).

### 4.8. ELISA Assays

Concentrations of CXCL10, IFNβ and IFNγ in KERTr medium were quantified by ELISA assays (DuoSet ELISA Development System, R&D Systems, Minneapolis, MN, USA). All procedures were performed strictly according to the producer’s instructions. All measurements were conducted in duplicate. The reaction was developed using a TMB solution (eBioscience, Thermo Scientific) and stopped by sulfuric acid. Optical density was recorded at a 450-nm wavelength (with correction to 570 nm).

### 4.9. Statistics

Data are presented as the means ± SD of the results from at least three independent experiments. Comparisons between two groups were analyzed using a two-tailed Student’s *t*-test. Significance was assumed at *p* < 0.01. The median absolute deviation (MAD) test was employed to detect outliers.
